# Estimated and in vivo measurements of bite force demonstrate exceptionally large bite forces in parrots (Psittaciformes)

**DOI:** 10.1111/joa.14144

**Published:** 2024-09-24

**Authors:** Shannon L. Harrison, Gregory P. Sutton, Anthony Herrel, D. Charles Deeming

**Affiliations:** ^1^ School of Natural Sciences, University of Lincoln Joseph Banks Laboratories Lincoln UK; ^2^ Département Adaptations du Vivant, Bâtiment d'Anatomie Comparée UMR 7179 C.N.R.S/M.N.H.N. Paris France; ^3^ Department of Biology, Evolutionary Morphology of Vertebrates Ghent University Ghent Belgium; ^4^ Department of Biology University of Antwerp Wilrijk Belgium; ^5^ Naturhistorisches Museum Bern Bern Switzerland

**Keywords:** allomtery, bite force calculation, feeding, in vivo, muscle dissection, parrots, physiological cross‐sectional surface area

## Abstract

Jaw morphology and function determine the range of dietary items that an organism can consume. Bite force is a function of the force exerted by the jaw musculature and applied via the skeleton. Bite force has been studied in a wide range of taxa using various methods, including direct measurement, or calculation from skulls or jaw musculature. Data for parrots (Psittaciformes), considered to have strong bites, are rare. This study calculated bite force for a range of parrot species of differing sizes using a novel method that relied on forces calculated using the area of jaw muscles measured in situ and their masses. The values for bite force were also recorded in vivo using force transducers, allowing for a validation of the dissection‐based models. The analysis investigated allometric relationships between measures of body size and calculated bite force. Additionally, the study examined whether a measure of a muscle scar could be a useful proxy to estimate bite force in parrots. Bite force was positively allometric relative to body and skull mass, with macaws having the strongest bite recorded to date for a bird. Calculated values for bite force were not statistically different from measured values. Muscle scars from the adductor muscle attachment on the mandible can be used to accurately predict bite force in parrots. These results have implications for how parrots process hard food items and how bite forces are estimated in other taxa using morphological characteristics of the jaw musculature.

## Graphical abstract

Bite force in parrots has been explored using data from muscle masses. The values were validated by comparison with data for bite force collected from live parrots. The parrot's reputation for a strong bite force is well founded.

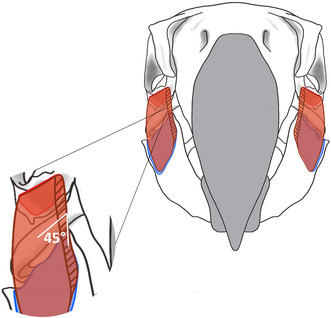

## 1 INTRODUCTION

The jaw is a functional unit which represents a direct interaction between the skeletal and muscular system of an organism. Bite force is heritable (Anderson et al., 2008; Zablocki‐Thomas et al., 2021) and is a good indicator of an animal's dietary and behavioural ecology (Aguirre et al., 2003; Herrel et al., 2001; Krishnan, 2023; Maestri et al., 2016; Nogueira et al., 2009; Sakamoto, 2021). As such, the biting capability (bite force) of an animal has been widely studied in vertebrates (Sakamoto et al., 2019), ranging from fish (e.g., Herrel et al., 2002), amphibians (e.g., Deban & Richardson, 2017), reptiles (reviewed by Deeming, 2022), birds (reviewed by Deeming et al., 2022), and mammals (Grandal‐d'Anglade, 2010; Grubich et al., 2012; Holliday, 2009; Mazzetta et al., 2009; Thomason, 1991; Wroe et al., 2005). More recently, several studies have also measured mandible forces in invertebrates, such as ants (Püffel et al., 2021).

Bite force is often measured in vivo with vertebrates biting down on force transducers (Ellis et al., 2008; Herrel et al., 1998; Sustaita & Hertel, 2010; Verma et al., 2017). However, in instances where this is not possible, for example, when specialised equipment is not available, or for extinct species in a palaeontological context, bite force can be inferred through the use of skeletal elements, such as skull width (Anderson et al., 2008) and skull length (Wroe et al., 2005) in mammals, or, in dinosaurs, dimensions of the cranial adductor chambers (Mazzetta et al., 2004; Rayfield et al., 2001; Sakamoto et al., 2019). Alternative methods include calculation of bite force following finite element analysis of 3D scans derived from computerised tomography (e.g., Cost et al., 2020).

Alternatively, heads can be dissected to quantify muscle masses, which can be used to calculate the physiological cross‐sectional area (PCSA) of the muscles, which, using a value for muscle fascicle length, and in combination with skull morphology, can be used to calculate bite force (e.g., Carril et al., 2015; Soons et al., 2015; Sustaita, 2008). A muscle's PCSA has been found to be proportional to the amount of force in which it can generate, assuming constant stress of the muscle fibres during contraction (Dickinson et al., 2022; Medler, 2002). The physical properties of muscle, that is, its mass and the configuration of the muscle fascicles, can also influence the PCSA (Püffel et al., 2023). For example, some muscles have fascicles which are arranged parallel to one another, following the muscles line of contraction (otherwise known as the line of action; LOA) (Demuth et al., 2022) or at variable pennation angles (Sullivan et al., 2019). However, muscles within the jaw are typically much more complex (Grünheid et al., 2009; Sharp & Trusler, 2015), with fascicle arrangements which are typically found at angles to the LOA (Cost et al., 2020). Moreover, physical measurements of fascicle length can be quite difficult to attain, particularly in the deeper muscles, such as the *ethmomandibularis* in parrots, where physical dissections can lead to distortions within the tissue which can affect any measurements taken (Dickinson et al., 2018). More recent works have attempted to circumnavigate this problem via the use of micro‐CT to visualise the muscles in situ (Holliday et al., 2022; Jeffery et al., 2011; Katzke et al., 2022). However, whilst some studies use multiple methods to confirm calculations (e.g., Soons et al., 2015) in many cases the validity of the calculated bite forces has not been compared against empirical values (but see Sakamoto et al., 2019), which is often due to methodological constraints, that is, possession of a force transducer or access to the animal of interest (Dickinson et al., 2022).

How bite force scales with body mass has been investigated in various species. In all studies to date, bite force exhibits positive allometry with body mass, for example, in finches (Fringillidae), various lizards, and piranhas (Serrasalmidae) (D'Amore et al., 2018; Meyers et al., 2002; van der Meij & Bout, 2004; Velasco‐Hogan & Meyers, 2021). Additionally, predicted bite forces scaled positively with body mass for a wide range of mammal predators, and in bats (Aguirre et al., 2002; Meers, 2002). Bigger species, irrespective of taxon, seem to be associated with greater bite forces. The morphological traits that underpin these relationships almost certainly reflect skull morphology but will have consequences for ecology, including diet.

Despite the interest in the factors affecting bite force in mammals and reptiles (Sakamoto et al., 2019) there has been relatively little interest in determining bite force in birds (but see Corbin et al., 2015; Deeming et al., 2022). This is curious given the interest in understanding the factors driving the evolution of diversity in beak morphology (Bright et al., 2019; Cooney et al., 2017; Navalón et al., 2019). Previous investigations of bite force in birds have focussed on the biting capability of Falconiformes (Hull, 1991; Sustaita, 2008), and various passerines (Herrel et al., 2005a, 2005b; Rao et al., 2018; van der Meij & Bout, 2004), which probably reflects the specialised mode of feeding in the species selected. Relatively few other bird species have been studied; data are reported for only 77 avian species compared with 170 species of non‐avian reptile (Deeming, 2022; Deeming et al., 2022). An analysis by Deeming et al. (2022) suggested that there may be a distinction between non‐passerines and passerines in the allometric relationships between body size and bite force but not between jaw muscle mass and bite force. However, it was concluded that the low number of species represented hampered our understanding of the functional properties of the jaw musculature in birds.

Whilst renowned for having strong bites (King et al., 2015; Navalón et al., 2019; Sakamoto, 2021) there has surprisingly been relatively little study into the biting mechanics of Psittaciformes. Bite force has been measured in rosy‐faced lovebirds (*Agapornis roseicollis*) using a force plate (Dickinson et al., 2022) and calculated for two other parrot species, from muscle dissection in the Monk parakeet (*Myiopsitta monachus*; Carril et al., 2015), and from digital dissection using computerised tomography in the African grey parrot (*Psittacus erithacus*; Cost et al., 2020). The bite force values for these three species of 18.3, 16 and 63 N, respectively, seem rather low for these small to mid‐sized species (body mass range ~ 50–333 g; Dunning Jr, 2008). For instance, estimated bite force in the large ground finch (*Geospiza magnirortis*), is 65 N, which is similar to that of the African grey parrot, despite the finch being 10 times smaller in mass (Soons et al., 2015). Given the well‐muscled jaw apparatus of these parrots (Carril et al., 2015; Homberger, 2017), and the damage a parrot bite can inflict (King et al., 2015), we wanted to investigate whether previous reported bite forces are representative of parrots. Parrots have similar muscle groups to other birds (Harrison et al., 2022) but have an additional adductor muscle, the ethmomandibularis (Burton, 1974; Bühler, 1981; Carril et al., 2015; Cost et al., 2020; Homberger, 2017; Tokita, 2003), which is the heaviest muscle in the jaw of the Monk parakeet and contributes the greatest force of the muscles (Carril et al., 2015). Presumably having an additional retractor muscle (see Deeming et al., 2024) would increase bite force? Furthermore, by studying various parrot species, we would be increasing the dataset for bite force reported for birds.

Here, we provide bite force estimates for a range of parrot species of differing size using a novel method that relies on the area of jaw muscles measured in situ and their masses to determine the forces generated by the muscles. The analysis allowed for comparison of allometric relationships between body mass, skull dimensions, jaw muscle mass, and calculated bite force. In addition, force transducers were used to measure bite force in situ for a range of parrot species allowing comparison with calculated values based on anatomy. Specifically, we tested the following hypotheses: (1) there will be isometric relationships between bite force and body mass, skull size, or jaw muscle mass and (2) when controlling for body mass and phylogeny calculated bite forces will be similar to in vivo forces measured using a force transducer. Given the practical complexity of calculating bite force from dissections, we also explored whether a simple relationship between the scar left by the *m. adductor mandibulae externus* and the bite force was useful in predicting bite force from parrot skulls. Therefore, our third hypothesis was that there will be a positive relationship between bite force and the length of the residual attachment scar.

## 2 METHODS

### 2.1 Dissection and specimen preparation

Parrot cadavers were supplied by the Lincolnshire Wildlife Park, Friskney, Lincolnshire, UK. All birds had died of natural causes and sample sizes varied according to species (Table 1). Due to potential effects of ill health prior to death, body mass values were retrieved from Dunning Jr (2008). The birds were stored in plastic bags and frozen at −20°C until required, when they were defrosted overnight at room temperature.

**TABLE 1:** Mean (±SE) values for body mass (in g, as reported by Dunning Jr, 2008), skull mass (in g), skull length (in mm), total jaw muscle mass on one side (in g), and calculated bite force (N) for each parrot species in three different families as indicated.

Species	Family	Body mass (g)	Skull mass (g)	Skull length (mm)	Total jaw muscle mass (g)	Bite force (N)	Bite force:Body mass ratio
*Cacatua alba* (*N* = 3)	Cacatuidae	570.0	12.6 ± 1.4	78.0 ± 2.2	4.8 ± 0.7	180.4	0.316
*Cacatua galerita* (*N* = 3)	Cacatuidae	720.4	12.0	76.9	6.8	167.3 ± 24.2	0.232
*Cacatua moluccensis* (*N* = 1)	Cacatuidae	835.0	18.7	91.3	6.6	264.7	0.317
*Melopsittacus undulatus* (*N* = 1)	Psittaculidae	29.0	0.4	27.3	0.1	12.4	0.428
*Psittacula eupatria* (*N* = 1)	Psittaculidae	214.0	5.0	59.0	2.7	105.7	0.480
*Psittacula krameri* (*N* = 4)	Psittaculidae	116.1	2.0 ± 0.0	45.8 ± 0.9	0.7 ± 0.1	34.6	0.298
*Ara ararauna* (*N* = 3)	Psittacidae	1125.0	20.4 ± 1.0	106.4 ± 5.3	7.1 ± 0.7	201.1 ± 57.6	0.179
*Ara chloropterus* (*N* = 3)	Psittacidae	1214.0	35.6 ± 3.6	117.8 ± 1.5	11.8 ± 1.2	374.9 ± 46.6	0.309
*Ara macao* (*N* = 1)	Psittacidae	1015.0	31.5	103.9	6.5	443.8	0.437
*Amazona aestiva* (*N* = 2)	Psittacidae	451.0	6.4	65.6	1.8	117.7	0.261
*Amazona amazonica* (*N* = 3)	Psittacidae	370.0	6.6 ± 1.0	67.6 ± 2.0	1.8 ± 0.1	83.1 ± 8.1	0.225
*Amazona auropalliata* (*N* = 1)	Psittacidae	476.9	6.5	65.6	2.4	93.6	0.196
*Amazona autumnalis* (*N* = 2)	Psittacidae	416.0	5.7	72.2	2.0	84.9	0.204
*Amazona farinosa* (*N* = 1)	Psittacidae	626.0	9.7	79.8	3.3	136.1	0.217
*Amazona oratrix* (*N* = 1)	Psittacidae	517.0	9.6	72.4	1.5	123.0	0.238
*Amazona ochrocephala* (*N* = 3)	Psittacidae	476.9	5.5 ± 0.3	65.3 ± 2.2	2.1 ± 0.2	77.3 ± 7.1	0.162
*Myiopsitta monachus* (*N* = 3)	Psittacidae	120.0	1.3 ± 0.2	41.8 ± 2.8	0.5 ± 0.0	21.3 ± 2.4	0.178
*Poicephalus senegalus* (*N* = 2)	Psittacidae	147.0	2.7	49.6	1.0	34.8	0.237
*Psittacus erithacus* (*N* = 5)	Psittacidae	333.0	6.8 ± 0.5	71.5 ± 1.8	2.7 ± 0.4	84.1 ± 6.2	0.252

*Note*: Number of individuals in the dataset are indicated in parenthesis. Standard error values were calculated for species where there were 3 or more individuals present.

As described in Harrison et al. (2022) the heads of the parrots were removed from the bodies before being skinned, exposing the muscles. Heads were placed and supported on their side to keep the lateral face of the head parallel to the table surface. Digital images were taken sequentially before, during and after the dissection using a Pentax X50 digital camera (Ricoh, Japan). Each muscle was identified, dissected away from the skull, blotted dry and the wet mass recorded using a digital Sartorius® micro balance. For all dissections, the scale bar was placed behind the head and the images were taken from a distance of 300 mm from a lateral viewpoint. In some specimens, the muscles on one side of the head were damaged, but in most cases, both sides of the head were dissected. The data presented are for an average muscle mass on one side (see Table 1).

Following dissection, the skull was de‐fleshed using the method described by Harrison et al. (2022) using Tergazyme at 15% concentration at 40°C. The skulls were then left for 2–3 days to dry in an incubator at room temperature. Each skull was weighed after drying using an electronic balance and the total length was measured with digital callipers from a dorsal view and was the distance between the supraoccipital crest and the tip of the maxilla.

The measurements of a muscle attachment scar along the *m. adductor mandibulae externus* were also taken post dissection using scaled laterally arranged images of the de‐fleshed and museum specimens (Natural History Museum, Tring) skulls in ImageJ (Schneider et al., 2012). Due to two different bite force estimates being suggested within this manuscript, bite force values determined from the *m. adductor mandibulae* scar will be referred to as the predicted bite force and force values generated from physical dissection and biomechanical modelling (outlined below) will be referred to as calculated bite force.

### 2.2 Biomechanical modelling

The jaw was treated as a static third‐class lever in which the biomechanical models were viewed in a 2D setting. Measurements of the images were taken using ImageJ (Schneider et al., 2012). It should be noted that this is a static model whereby the effects of cranial kinesis were not considered given that the extent to which the upper beak can move relative to the skull is highly dependent on active manipulation of the jaw muscles.

**FIGURE 1:**
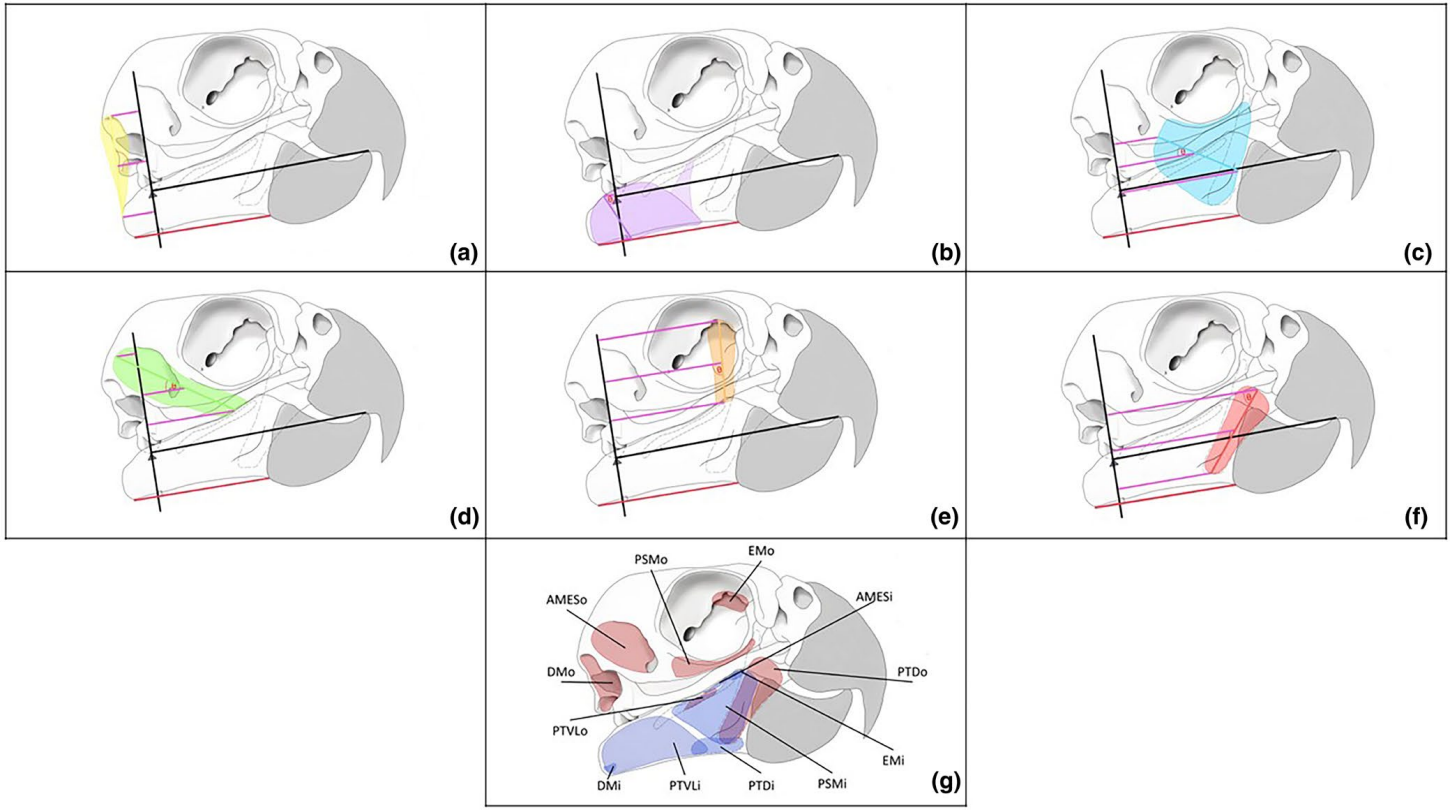
An illustrative outline of an *Cacatua alba* skull used to measure bite force per muscle: Yellow (a): *m. depressor mandibulae* (DM), purple (b) = *m. pterygoideus ventralis* (*PTVL*), green (c) = *m. adductor mandibulae* (*AME*), blue = *m. pseudomasseter* (*PSM*), red (e) = *m. pterygoideus dorsalis* (*PTD*) orange (f) = *m. ethmomandibularis* (*EM*). Panels b–f represent the abductor and adduction muscles which were used in presented bite force calculations. Filled in coloured regions indicate the surface area of the muscle and corresponding line colours the moment arms (pink lines) extending from the perpendicular bisect from the fulcrum (black line). Solid lines within the muscles (of corresponding colour) indicate the line of action (LOA). Dashed lines represent the bony structures which are situated behind the lower jaw: From left to right, lower element of the quadrate, *os pterygoideus* and palatine bone. Red horizontal line = established horizontal from which relative measurements were taken, Black vertical line = Moment arm out (Mout). All moment arm measurements ran parallel to the defined horizontal. Panel G maps the origin (o, red coloured regions) and insertion (i, blue coloured region) of the muscles defined through a–f. Dashed yellow line (panel g) represents the *m. adductor mandibulae externus* scar.

Multiple images of different dissection stages were taken to account for arrangement of the muscle in situ on the skull when taking moment arm and surface area measurements. Whilst it was necessary to use different images of the stages of dissection, this potentially introduced some error due to the re‐positioning of the specimens. Therefore, it was necessary to establish a controlled point of reference which could be measured between images and specimens. The points between the fulcrum and the posterior edge of the rhamphotheca were treated as the horizontal from which angular measurements were taken (Figure 1). The base of the quadratojugal‐quadrate complex process was treated as the fulcrum (represented by the pink triangle in Figure 1).

The line of action (LOA) for each muscle was colour coded and drawn on the image and then lines from the muscle force vector towards the perpendicular bisect drawn although the fulcrum. To calculate the amount of force the jaw muscles can generate, the moment of force was measured per muscle. However, since the muscles were not uniform rectangles and different parts of the muscle had different lengths from the origin to insertion, a mean moment arm was calculated per muscle, therefore considering the average distance of the muscle from the point of pivot. These moment arms (pink lines in Figure 1) were measured as the orthogonal distance to the perpendicular bisect (black line through the fulcrum in Figure 1). Two measurements were taken from both ends of the LOA, effectively acting as a ‘start and end’ point of the muscle, and an additional moment arm was drawn midway between these two points. From these three measurements, an average moment arm was calculated.

Angles to the horizontal and surface area of each muscle were measured using ImageJ. In the case of the *m. pterygoideus dorsalis* whereby the entire muscle body was not visible, the area of palatine bone was used as a proxy for surface area because this muscle exhibits surface attachment along this bone.

### 2.3 Physiological cross‐sectional area estimate

Previous work used the fascicle length to calculate physiological cross‐sectional area (PCSA; Carril et al., 2015; Sustaita, 2008) but there is ambiguity in the literature relating to what constituted fascicle length because it has also been referred to as fibre length (Gusekloo & Bout, 2005; van der Meij & Bout, 2004). Following Taylor et al. (2018), we treated the fascicle as the distance from the central muscle tendon to the terminating edge (the most distal point of the muscle, relative to the central tendon). For a specimen of *P. erithacus*, muscles were dissected and isolated before being soaked in 70% ethanol solution and viewed under a dissection microscope (VWR®) between 10 and 20× magnification depending on muscle size. Images were taken of these muscles using a phone camera (Apple, 2018) connected to the microscope ocular lens. Ten fascicles were removed from each muscle and measured using ImageJ and an average fascicle length was calculated. However, it was noticed that estimated PCSA calculations based on this value were still relatively low (around 16 N) in comparison with other calculated data and for predicted models. For example, *P. erithacus* has an average jaw muscle mass of 2.7 (±0.4) g, which under the prediction outlined by Deeming et al. (2022) should have a minimum bite force of around 42 N. Moreover, Cost et al. (2020) used finite element modelling to predict a bite force range of 62–96 N for this species.

**FIGURE 2:**
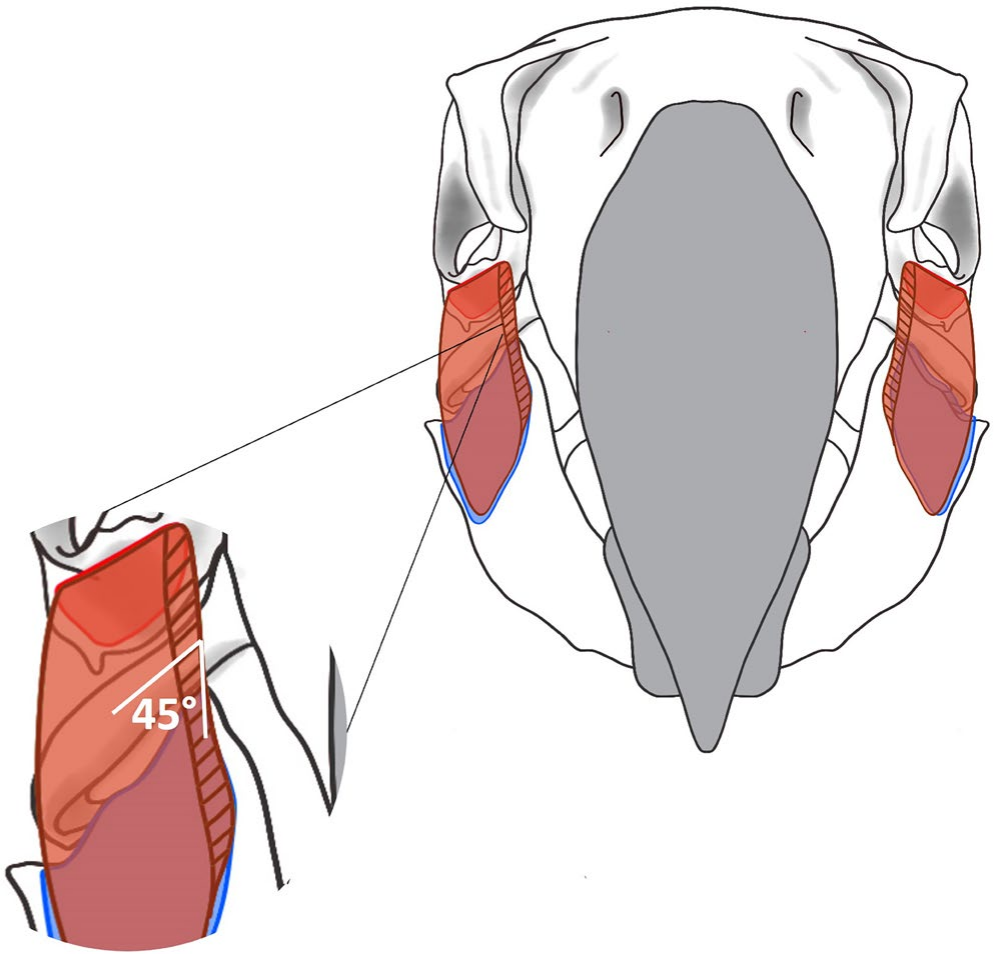
An illustrative outline viewed anteriorly demonstrating the proposed attachment of muscle between the skull and the mandible. The depicted muscles is the *m. pseudomasseter* which originates at the zygomatic arch (red region) and inserts laterally along the surface of the mandible (blue region). The opaque dark red region indicates muscle, and dark red lines depict the fascicles at 45° (magnified), viewed in cross section anteriorly.

Whilst individual fascicle dissection was unsuccessful, a new methodology was generated in which the thickness (cross sectional width) of the muscle was calculated using both the muscle surface area and calculated muscle volume. The surface area of each muscle was measured (measurements were taken from the step‐wise dissection process) and then the volume of each muscle was calculated by dividing the mass by a standard density of muscle tissues of 1.06 mg/mm^3^ (Carril et al., 2015; Sustaita, 2008). These two elements were combined using the formula:

(1) T=VA,

where *T* = muscle thickness, *V* = muscle volume and *A* = muscle area. This gave the thickness of each muscle if each fascicle was projected outwards away from the bone (as seen in leaf cutter ants *Atta vollenweideri*; Püffel et al., 2021). However, visual inspection of the fascicles during dissection indicated that they were more densely packed, originating from the bony elements at an angle. Therefore, the thickness values were divided by sin 45° to get the fascicle length, angled from the bony elements (see Figure 2). Pennation angle of the fibres was based on the micro‐CT measurements of *P. erithacus* by Cost et al. (2020).

It was noted that the measurements taken from ImageJ and the physical measurements, for example the distance between fulcrum and mandible tip, were providing different values. It is believed that this discrepancy came from lens compression (Banks et al., 2014). This was corrected for by comparing the distance between two known points between the physically and digitally measured distances and then calculating a scale factor. This scale factor for each specimen was then used to adjust the surface area measurements prior to calculating PCSA.

PCSA (mm^2^) was calculated following (Carril et al., 2015; Sustaita, 2008):

(2) $$\text{PCSA}\;\left({\mathrm{mm}}^{2}\right)=\frac{\left(m\times \cos\theta \right)\;}{\left(\rho \times l\right)}$$

where *m* = mass of the muscle, *θ* = angle (in radians) of pennation following the measurements from the African grey parrot reported by Cost et al. (2020), ρ = density of the muscle (1060 g/mm^3^) and *l* = fascicle length as defined above. Muscle force (*F*) was calculated for each muscle by multiplying the PCSA by 0.3 N/mm^2^, a common estimate of tetanic stress in muscles (Medler, 2002). Torque (*T* in N/mm) generated by each muscle was calculated:

(3) Τ=rFsinθ

where *r* (radius) = average moment arm length (mm), *F* = calculated muscle force (N), and sin*θ* = angle of line of action to the defined horizontal. Total torque, that is, the sum of the torque generated by each muscle, was then divided by the out‐lever moment arm (mm) and multiplied by two to give the overall bite force (N). The duplication provided the bite force for both sides of the jaw. The forces generated by each of the adduction muscles (see Figure 1) were summed to determine the overall force generated by the jaw musculature.

### 2.4 Empirical measurement of bite force

Bite forces were recorded for various captive parrots at zoos and animal rescue facilities in Florida, USA as part of a master thesis project (Table 3; Jaffe, 2007). Bite forces were measured with a Kistler piezo‐electric force transducer (type 9203, range ±500 N and 9311B, range ±5000 N; Kistler Inc., Winterthur, Switzerland) mounted in a custom‐built holder, and connected to a portable Kistler charge amplifier (type 5059A) as described previously (Herrel et al., 1998, 2005a, 2005b). The bite plates were moved farther apart when testing larger animals to ensure that all birds were biting at equivalent gape angles (30°). Bite plates were modified from the standard flat plates to rounded plates that fitted the beak of the parrots better and covered with medical tape to prevent damage and provide grip. Several bites were recorded for each individual and the strongest bite was used in subsequent analyses as an estimate of maximal bite force.

### 2.5 Statistical analyses

Measurements for all values were log_10_‐transformed prior to analysis. Analyses to compare morphological measurements, that is, body mass, skull mass, skull length, jaw muscle mass and calculated bite force, were performed in R (version 4.0.3; R Core Development Team, 2021) using the statistical packages “*ape*” (Paradis et al., 2004), “*MVTnorm*” (Genz & Bretz, 2009), and “*MASS*” (Venables & Ripley, 2002), with additional code supplied by Dr. Carl Soulsbury to run the phylogenetically controlled generalised linear models. To control for phylogeny, a time‐calibrated phylogeny was constructed using a subset of species downloaded from birdtree.org (Jetz et al., 2012). This tree was loaded into the R environment using the package “*phytools*” (Revell, 2012). One‐sample *t*‐tests were used to compare derived exponents against expected values (Bailey, 1981).

Calculated and predicted bite forces were compared against the measured bite forces using a Bayesian approach to control for the degree of relatedness in the relationships where there were values for individual species derived from different sources. The model was run in R using the packages “*MCMCglmm*” (Hadfield, 2010), “*Matrix*” (Bates et al., 2015) and “*phytools*.” The phylogenetic tree was derived as above. In this model, the settings included parameter expanded uninformative priors, 500,000 independent chain iterations, which were sampled every 500 iterations after a 10,000 burn in. The initial model included body mass as a covariate (Figure 6) and type of measurement (i.e., measured versus calculated) as a categorical factor and included an interaction term. If the latter was not significant, the model was simplified and re‐run. Lambda (*λ*, which is the phylogenetic signal) was calculated by dividing the variance explained by the phylogeny, by the sum of all variance components. Lambda varies from 0 (little to no covariance in the residuals due to phylogeny), to 1 which indicates that variance in the residuals is because of shared ancestry expected under a Brownian model of trait evolution (Freckleton et al., 2002).

## 3 RESULTS

Average calculated bite force values ranged from 12 to 444 N with the smallest values for the Budgerigar (*Melopsittacus undulatus*) and the largest bite force value being for the Scarlet macaw (*Ara macao*; Table 1). The phylogenetically controlled relationship between body mass and bite force was highly significant, explaining over 88% of the variation in the data (Table 2). The phylogenetically controlled slope of body mass to bite force was not significantly different from an isometric slope of 0.66 (*t*_18_ = 0.632, *p* > 0.05) and the phylogenetic signal was moderately weak at 0.39.

**TABLE 2:** Results from linear and phylogenetically corrected linear regression models testing the relationships between calculated bite forces (N), average body mass (g), average skull mass (g), average skull length (mm) and average jaw muscle mass (g).

Relationship modelled	Phylogenetically controlled general linear model			
	Exponent (SE)	*t* (*p*‐value)	*R* ^2^	*λ*
Log Bite force ~ Log Body mass				
Intercept	−0.503 (0.229)	−2.19 (<0.042)		
Slope	0.978 (0.087)	11.20 (<0.001)	0.88	0.39
Log Bite force ~ Log Skull mass				
Intercept	1.318 (0.045)	29.15 (<0.001)		
Slope	0.828 (0.042)	19.78 (<0.001)	0.95	0.29
Log Bite force ~ Log Skull length				
Intercept	−2.706 (0.352)	−7.69 (<0.001)		
Slope	2.574 (0.191)	13.45 (<0.001)	0.91	0.24
Log Bite force ~ Log Jaw muscle mass				
Intercept	1.538 (0.052)	29.03 (<0.001)		
Slope	0.715 (0.065)	11.02 (<0.001)	0.88	<0.001
Loge Bite force ~ Low Adductor scar length				
Intercept	−0.026 (0.166)	−0.16 (0.875)		
Slope	2.195 (0.178)	12.33 (<0.001)	0.90	<0.001

**FIGURE 3:**
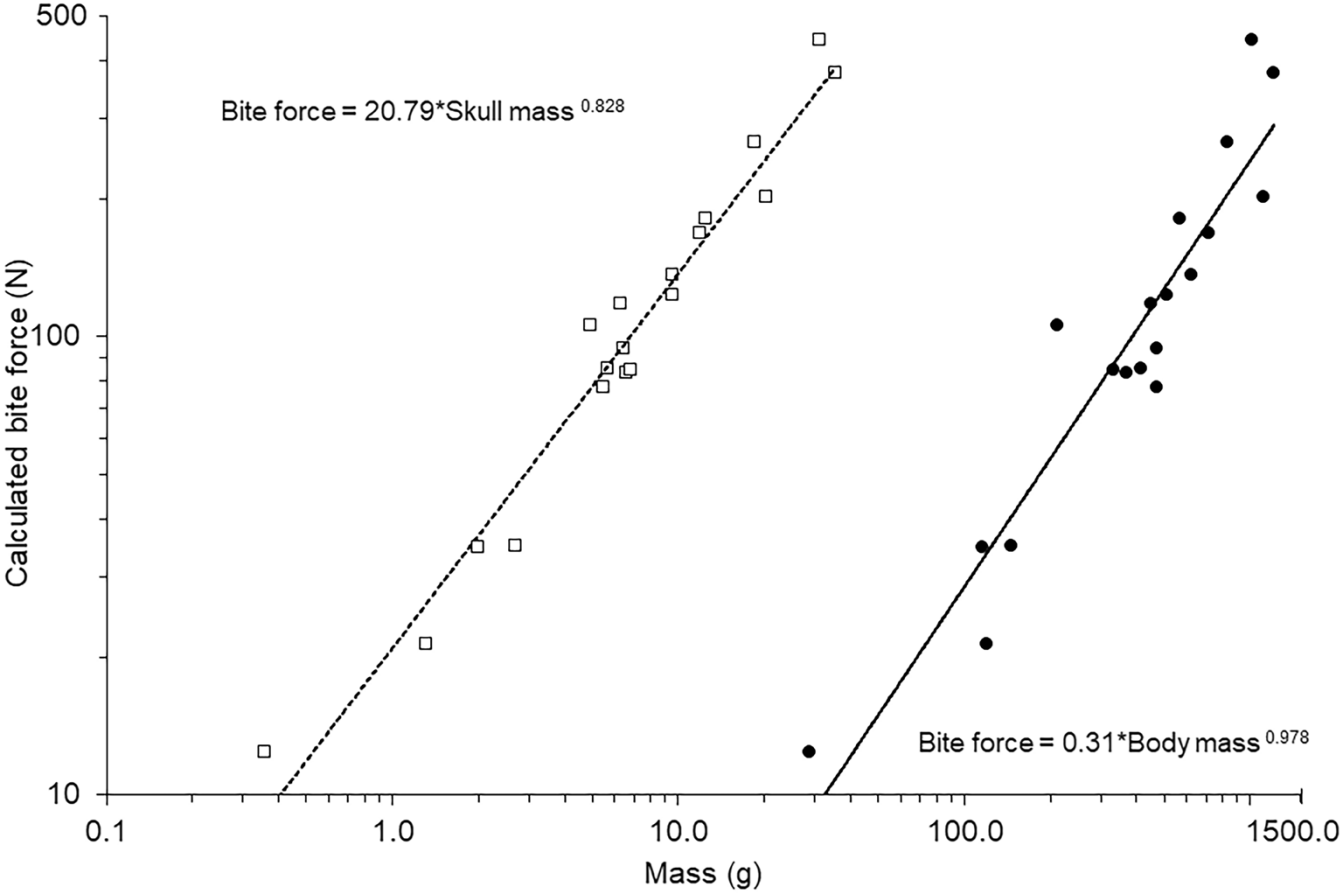
Relationships between average values for calculated bite force and skull mass (open squares) and body mass (Dunning Jr, 2008) (solid circles). Trendline and equation indicate the phylogenetically controlled general linear model generated in R.

**FIGURE 4:**
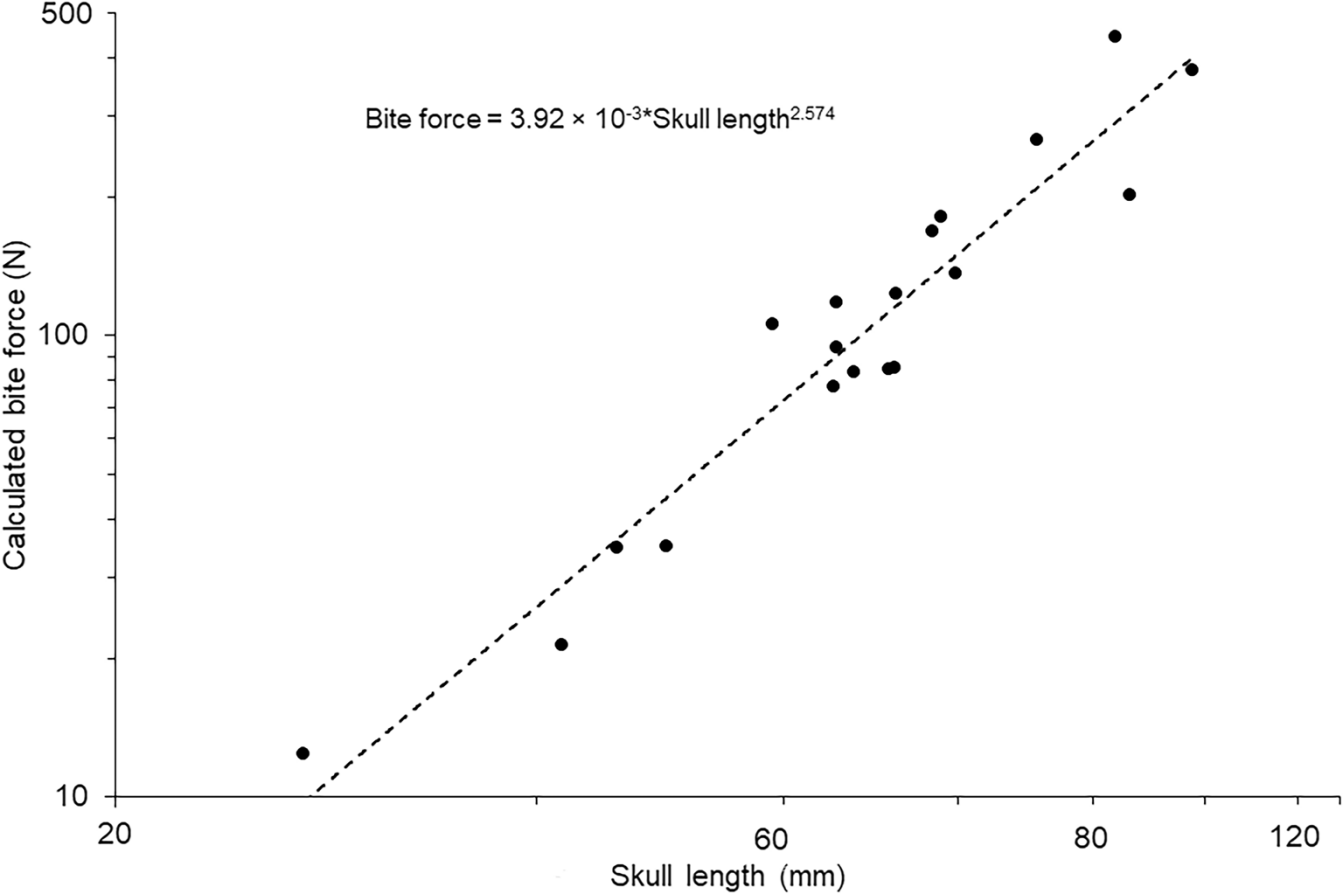
Relationships between average values for calculated bite force and skull length (solid circles). Trendline and equation indicate the phylogenetically controlled general linear model generated in R.

The phylogenetically controlled relationship between calculated bite forces and skull masses was highly significant explaining around 95% of the variation in the data (Figure 3; Table 2). The slope of 0.83 was significantly different from the expected isometric slope of 0.66 (*t*_18_ = 4.03, *p* < 0.001) indicating positive allometry but with a low lambda value of 0.29. Skull length also had a highly significant positive relationship with calculated bite force values although lambda was low at only 0.24 (Figure 4; Table 2). There was a highly significant difference between the expected isometric slope of 2.0 and the phylogenetically controlled model slope of 2.57 (*t*_18_ = 3.01, *p* < 0.001) showed that this relationship was positively allometric.

**FIGURE 5:**
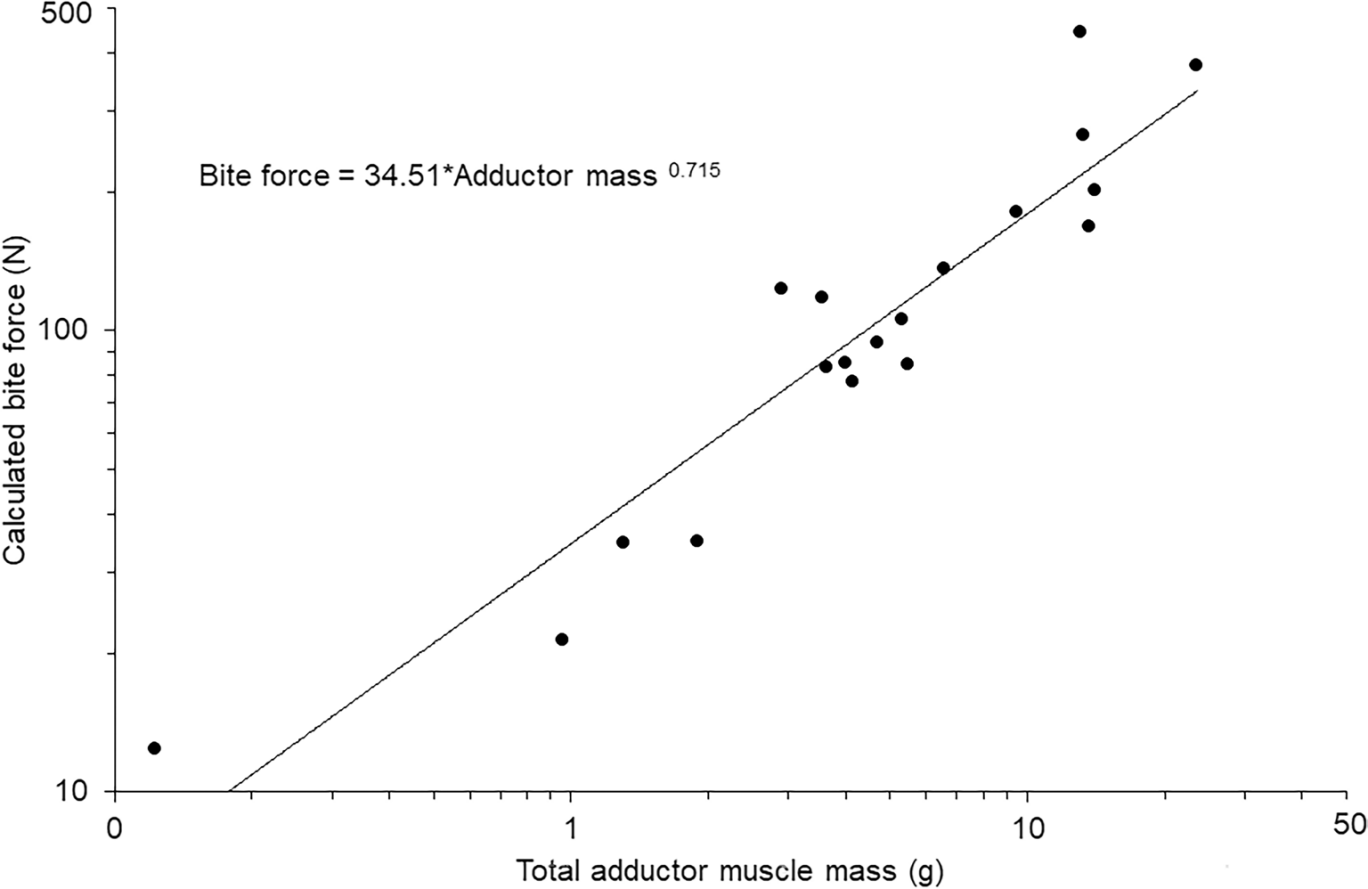
Relationships between average values for calculated bite force and total adductor muscle mass (solid circles). Trendline and equation indicate the phylogenetically controlled general linear model generated in R.

The sum of the muscle masses (Table 1) that contributed to bite force for each species had a highly significant positive relationship with calculated bite force values (Figure 5). Comparison of the phylogenetically controlled slope of 0.71 against an expected isometric slope of 0.66 indicated that jaw muscle mass scaled isometrically with calculated bite force values (*t*_18_ = 0.849, *p* > 0.05) and the lambda value for this relationship was very low (<0.001).

**FIGURE 6:**
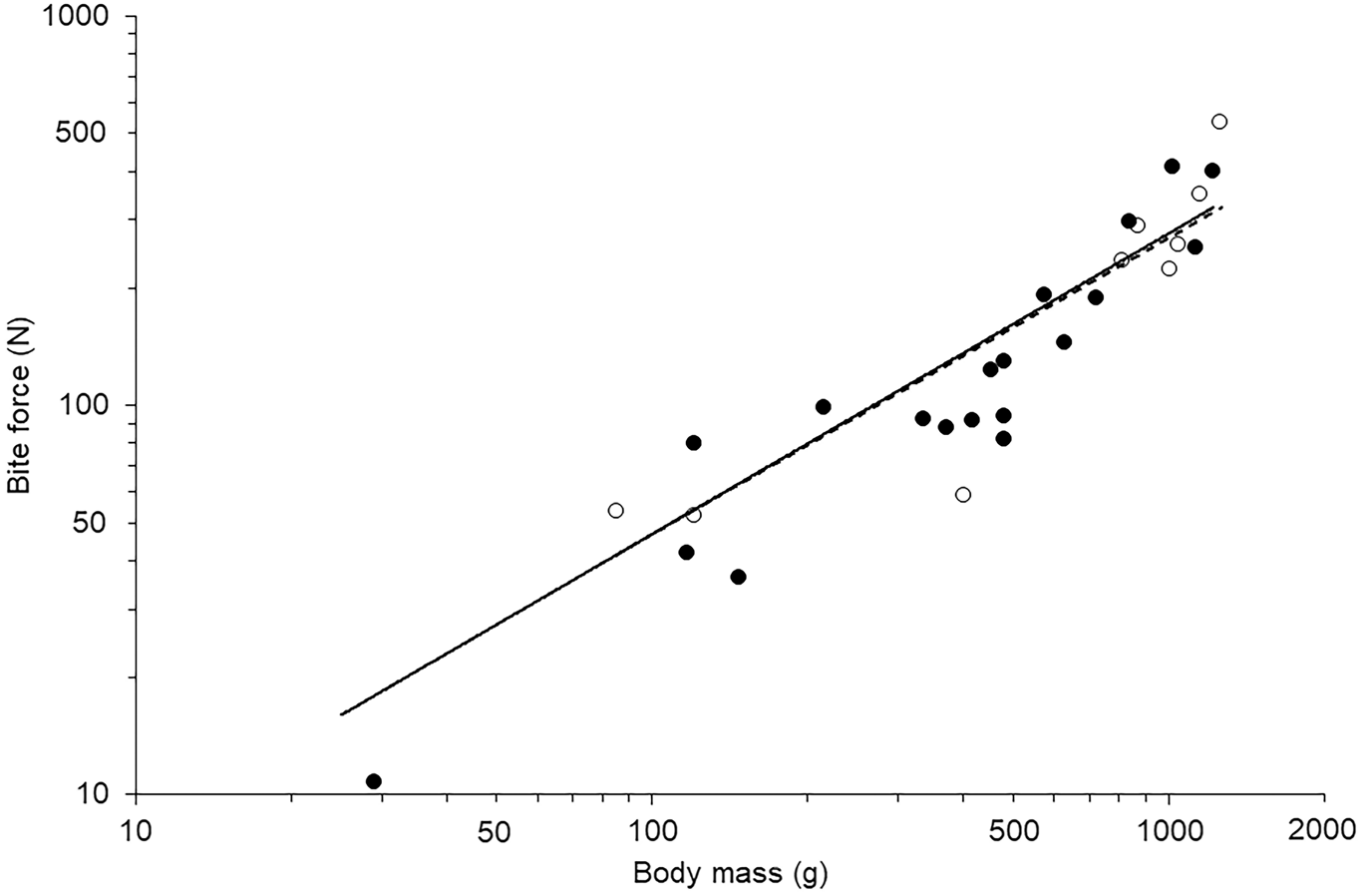
Relationship between body mass and bite force values for calculated (closed symbols) and measured (open symbols) forces. Trendlines indicates the individual linear relationships for the calculated (solid line) and measured values (dashed line) calculated by the phylogenetically controlled analysis in R.

Measured bite forces varied from 52.5 N for coconut lorikeet (*Trichoglossus haematodus*), that was a tenth of the mass of the hyacinth macaw (*Anodorhynchus hyacinthinus*), which had a bite force of 538.9 N, around ten times greater (Table 3). The values for calculated bite force and measured bite forces (Table 3) both exhibited positive relationships with body mass (Figure 6). Comparison of the two different measures of bite forces using a Bayesian phylogenetically controlled generalised mixed model, whilst controlling for body mass as a covariate showed no interaction between the covariate and the categorical factor (Posterior mean = −0.095; 95% credible intervals: −0.368 to −0.368; pMCMC_1158_ = 0.502). Therefore, the model was simplified to remove the interaction and whilst log body mass was highly significant (Posterior mean = 0.863; 95% credible intervals: 0.696 to 1.029; pMCMC_980_ < 0.001) there was no significant effect of the method of determining bite force (Posterior mean = −0.008; 95% credible intervals: −0.153 to −0.153; pMCMC = 0.947). The lambda was low at 0.105.

**TABLE 3:** Mean (±SE) body masses (BM) of nine species of parrots in which bite force (BF) was measured using samples sizes indicated.

Species	Body mass (g)	Measured bite force (N)	Measured BF:BM ratio	Predicted bite force (N)	Predicted BF:BM ratio
*Anodorhynchus hyacinthinus* (*N =* 7)	1253.7 ± 34.5	538.86	0.4298	466.81	0.3723
*Ara ararauna* (*N =* 13)	1040.2 ± 20.4	259.77	0.2497	209.78	0.2017
*Ara chloropterus* (*N =* 9)	1140.8 ± 28.9	350.22	0.307	393.44	0.3449
*Ara glaucogularis* (*N =* 1)	866	290	0.3349	n/a^b^	‐
*Ara macao* (*N =* 5)	1000.6 ± 71.4	224.8	0.2247	286.74	0.2866
*Ara militaris* (*N =* 5)	809.8 ± 71.2	237.7	0.2935	222.77	0.2751
*Ara severus* (*N =* 1)	400	59	0.1475	n/a^b^	‐
*Aratinga canicularis* (*N =* 1)	85^a^	53.6	0.6306	38.78	0.4562
*Trichoglossus haematodus* (*N =* 6)	120^a^	52.45	0.4371	23.97	0.1998

*Note*: Data are also provided for bite forces predicted from the measurements of the mandibular adductor scar on skulls from the same species (see Figure 6). The values for the appropriate bite force to body ratio are also included for measured and predicted values for bite force.

^a^ Mass values from Dunning Jr (2008).

^b^ No image of the skull was available for these species.

**FIGURE 7:**
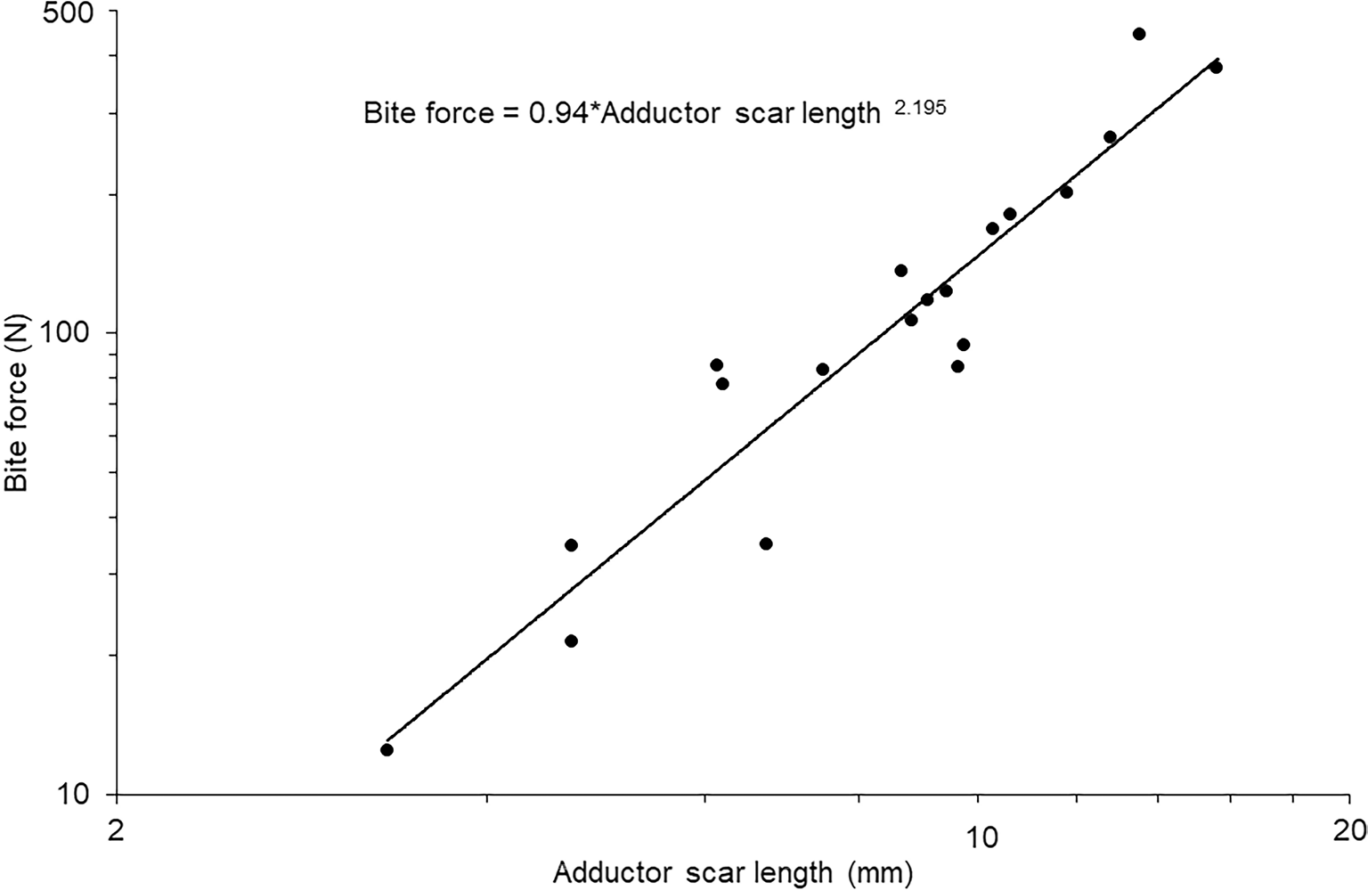
Relationship between *m. adductor mandibulae* scar length and calculated bite force values. Trendlines and equations indicate the phylogenetically controlled general linear models generated in R.

Calculated bite force values exhibited a strong linear relationship with the length of the residual scar left from the insertion of the *adductor mandibulae externus* complex (Figure 7). The phylogenetically controlled model explained around 90% of the variation in the data (Table 3) and the calculated bite force value exhibited isometry with the adductor scar length (Figure 7; *t*_18_ = 1.09, *p* > 0.05) and phylogenetic relatedness had no effect on this relationship (Table 2). This relationship between scar length and bite force had an *R* ^2^ value of (0.90) indicating that the scar length measurement can be used as a proxy for bite force.

Using the equation generated from the analysis above, that is,

$$\text{Predicted bite force}=0.94\;{\text{Adductor scar length}}^{2.195}$$

bite force values were estimated from skulls for the same species in which we had measured data (Table 3). Comparison of the predicted bite force estimated with the scar length and measured bite force values using a Bayesian phylogenetically controlled generalised mixed model, whilst controlling for body mass as a covariate, indicated no significant interaction (Posterior mean = −0.188; 95% credible intervals: −0.428 to 0.126; pMCMC_1150_ = 0.167). The simplified model (*λ* = 0.33) showed that log body mass was highly significant (Posterior mean = 0.926; 95% credible intervals: 0.588 to 1.249; pMCMC_980_ < 0.001) but there was no signification effect of the method of determining bite force (Posterior mean = 0.051; 95% credible intervals: −0.0.098 to 0.183; pMCMC_980_ = 0.947).

## 4 DISCUSSION

The results presented demonstrated that the relationships between bite force and body mass, and bite force and skull mass, were both positively allometric. The calculated values for bite force based on the measurements from dissection were not statistically different from values for bite forced measured from living birds. Scarring from the attachment on the mandible of the *m. adductor mandibulae externus* complex could be used as an osteological measure to accurately predict bite force in parrots.

### 4.1 Comparison with previous studies

Parrots have garnered a reputation for possessing a strong bite (Burton, 1974; Bright et al., 2019; Dickinson et al., 2022; King et al., 2015; Tokita, 2003). This study provides values for bite force for the highest number of parrot species so far and has increased the number of birds for which bite forces are recorded by ~25% (Deeming et al., 2022). Carril et al. (2015) reported a calculated bite force for the monk parakeet of 16 N, which was only two‐thirds of the value calculated here. However, Cost et al. (2020) estimated bite force in the African grey parrot using finite element analysis to be between 61 and 96 N (rostral to caudal positioning); the rostral estimate is 75% of the value calculated here.

More generally, the values for bite force reported for macaws are the highest yet recorded for any bird species (see review by Deeming et al., 2022). In this study, the highest measured bite force belongs to the Hyacinth macaw (*A. hyacinthinus*) with a bite force of almost 540 N. Outside of the group of Psittaciformes, the highest reported bite force values to date are 68.14 N for the large ground finch (*Geospiza magnirostris*), which weighs ~32 g (Soons et al., 2015), and 69 N for the king vulture (*Sarcoramphus papa*), which weighs 3375 g on average (Degrange et al., 2010). Of this study, only 4/19 parrot species have lower absolute bite forces than *G. maginirostris* and *S. papa* and these 4 species were relatively small (<147 g) for Psittaciformes.

However, that parrots have strong bites is not a side effect of their size. The ostrich (*Struthio camelus*) has a mass of 100 kg but a bite force of only around 50 N (Gusekloo & Bout, 2005). A female Cooper's hawk (*Accipiter cooperii*) has an average body mass of around 450 g and a measured bite force of 3.1 N (Sustaita & Hertel, 2010). By contrast, the blue‐fronted amazon (*Amazona aestiva*) also weighs 451 g but has a bite force of ~123 N. This supports the idea that parrots possess key morphological features that enable a high bite force (Carril et al., 2015; Tokita, 2003). However, in relation to their body mass, other birds have a stronger bite. Parrots have a bite force to body mass ratio ranging between 0.148 and 0.631 N/g, which are similar to values calculated for various finches (Fringillidae and Estrildidae) but some of the Darwin's finches (Thraupidae) have bite force to body mass ratio values exceeding 1.2 N/g (van der Meij & Bout, 2004).

### 4.2 Bite force and skull morphology

The relationship between body and skull mass in parrots was positively allometric, which could reflect disproportionately larger skulls being necessary as a response to the higher bite forces generated by bigger birds. For example, the higher bite forces transmit a higher level of stress on the supporting structures (the skull and ligaments) and this is reflected in a greater mass and size of the skull to better distribute the force during biting (Buckland‐Wright, 1978; Hart et al., 2017). Fry et al. (2009) reported that the relatively lightweight skull of the Komodo dragon (*Varanus komodoensis*) would be unable to withstand the high bite force generated by the Australian saltwater crocodile (*Crocodylus porosus*), which had a similar skull size. Therefore, skull architecture is also an important element to consider when studying bite forces because high forces cannot be generated if the supporting structure cannot withstand the stress.

During the dissections of the parrot heads, it was noted that in many species, such as White cockatoo (*Cacatua alba*) and the Blue‐and‐gold macaw (*Ara ararauna*), the lachrymal bone was fused with the post orbital process to form a solid suborbital arch (Zusi, 1993). By comparison, in other species, such as the Senegal parrot (*Poicephalus senegalus*), the post orbital process was not as well developed and terminated mid orbit leaving the ventral orbit rather undefined. Size of the birds was not a consideration here because medium sized *Amazona* sp. also had a solid suborbital arch. Therefore, these differences in skull morphology may reflect variation in diet (Navalón et al., 2019) or the degree of structural support required to distribute the bite force (Plateau & Foth, 2020; Tokita, 2003). The possible relationship between the presence of a sub‐orbital arch and the magnitude of the bite force in relation to diet is worthy of further study in a wider variety of parrots.

Interestingly, the length of the skull exhibited negative allometry with total jaw muscle mass. When coupled with the fact that there is positive allometry for skull length and bite force, it could be that the decrease in out‐lever length is compensating tor the disproportionally lighter muscles. A decrease in the out‐lever distance leads to the muscles having more mechanical advantage, so it also means that the amount of effort required to transmit force about the bill tip is reduced. This result suggests that there is a morphological trade‐off between the hard and soft tissues involved in jaw movement. The skull represents a finite amount of space, whereby multiple physiological factors are of importance (Sakamoto et al., 2019) and may serve as a more relevant scaling metric than body mass (Sustaita, 2008). Therefore, this trade‐off is likely reflective of these varying selection pressures influencing morphology of the skull and by association, the upper and lower beaks.

The greater the moment arm ratio between the length of the moment arm for the muscles and tip of the lower beak (the moment arm out), the greater the force able to be generated. Therefore, it could be possible that species with high bite forces, whilst not possessing a relatively high muscle mass may have developed a system in which this offset in mass is counteracted against by the disproportionately longer skulls in which the muscles are attached along the length of the lower jaw, further away from the fulcrum, thus increasing the force production. Additionally, the greater the distance between the fulcrum and the force generating muscles, the less effort is required by the muscles when active.

This translates into high bite forces by increasing the ratio between the lever arm of the force producing muscle and lever arm of the out force. Similar interactions between bite force and skull length have been reported in a range of carnivorous mammals (Anderson et al., 2008; Ellis et al., 2008; Thomason, 1991). For example, in domestic dogs (*Canis familiaris*, Canidae) bite force generally increased with skull length, but this increase was proportionately greater in short snouted dogs likely due to the shorter out lever arm transmitting a comparatively higher force in comparison to longer snouted breeds (Brassard et al., 2020). As in parrots, the higher bite force in shorter snouted dogs is likely because the muscles are conserved between breeds but shortening of the out‐lever increases the resultant bite force in comparison to a breed which has a similar muscle mass but longer snout.

The textbook example of a muscle is one that is long and thin with fascicles aligned in a long line that originate and insert on bones (one that is similar in geometry to the human *m. biceps brachii*). Muscle contraction then rotates bones around a joint (Gans, 1982; Lieber & Fridén, 2000). By contrast, jaw closing muscles in birds are mainly flat sheets that seem to suggest that the fibres are not aligned in long rows but rather are aligned to connect the aponeuroses that lie medially and laterally. In effect, the muscle is short and fat, with fibres placed such to maximize muscle force about the joint. Whilst the whole muscle may ultimately attach to often large areas of bone at both ends, the individual fibres are angled and have a wider range of movement (Brainerd & Azizi, 2005) so they connect the tendonous aponeurosis that connects the muscle to the bones with that on the distal aponeurosis that wraps around the muscle. Therefore, fibres in these muscles run from tendon to tendon and are angled such that when they contract the lateral aponeurosis is pulled laterally relative to the aponeurosis connected to the bone. This arrangement is best illustrated by the *m. pterygoideus ventralis* (PTVL) muscle that is attached to the lateral aspect of the mandible and connected by a tendon that runs under the ventral edge of the mandible. Its aponeurosis is continuous with that covers the muscle attached to the medial mandible and then on to ultimately insert on the pterygoid bone (Figure 1). Contraction of the muscle on the lateral side of the mandible can only pull on the surrounding aponeurosis, thereby pulling the medial tendon under the ventral mandible and so help bring the mandible and pterygoid closer.

Diffusible iodine‐based contrast‐enhanced computed tomography (diceCT) of jaw muscles can digitally isolate muscle fascicles but often their orientation does not necessarily match the line of action of the muscle. For instance, Jeffery et al. (2011) showed that the fascicles do not run parallel to the orientation of the deep masseter of the grey squirrel (*Scurius carolinensis*). In the crab‐eating macaque (*Macaca fascicularis*), fascicles of the posterior temporalis run contrary to the line of action of the muscle (Dickinson et al., 2018). It would be difficult to see how they would contribute any force to the muscle action unless they were connected to the overlying aponeurosis that is key to the muscle action. Thus, the arrangement of the parrot jaw closure muscles (large PCSA, large amounts of tendonous tissue, large pennation angles, and short fibres) (Azizi et al., 2008) is often seen in jaw closing muscles in other animals (including humans, van Eijden et al., 1997), and is characteristic of muscles that are oriented such that they maximize the force generated during behaviour (van Eijden et al., 1997).

Many studies emphasise that bite force cannot be considered in isolation from skull morphology (e.g., Fry et al., 2009). Many studies can measure bite force directly but many other are reliant on skull and jaw morphology to allow calculation of bite force but relatively few are able to validate calculations by comparison with empirical data. This study has highlighted a potential problem for jaw muscles based around the definition of the fascicle length, which is crucial to the calculation of PCSA and subsequently, bite force.

### 4.3 Bite force and PCSA

A major aspect of PCSA estimation is the use of the ‘fascicle length’ (Carril et al., 2015; Martin et al., 2020; Sustaita, 2008) but a definitive definition is hard to find in the literature. The term seems to be interchangeable, with, for example, muscle fibre length (Christiansen & Adolfssen, 2005; Narici et al., 1992; van der Meij & Bout, 2004). However, it is not possible to state with any certainty that in these instances whether the whole fibre of the muscle was measured rather than the individual fascicle lengths because these measurements were often not reported.

In the previous studies that have used this measurement for birds, the muscle fascicles are removed from the main muscle body and measured using a dissecting lens (Sustaita, 2008; Wang et al., 2017). Typically, the connective matrix which surrounds the fascicles is dissolved away using aqueous nitric acid or other solvents so as to expose the muscle fibre fascicle (Salzano et al., 2018; Sustaita, 2008). However, we observed that an excised muscle begins to shrink quickly and any attempt to isolate fascicles exacerbated this shrinkage. Other studies have fixed muscles in various solvents (Jones et al., 2019; Sellers et al., 2017; To et al., 2021) and then dissected them away from the skull after using a CT scanner to view the muscles in situ. Whilst this could be a preferred method, it still is restrictive in that CT scanners are relatively expensive and so are not always readily available.

Additionally, the solvents typically used in tissue preparation have been noted to cause differential changes at both a cellular and structural level (Buytaert et al., 2014; West, 2013). This partial shrinkage combined with the physical removal of the muscle can cause disruption of the muscle fascicles which may render any measurements inaccurate but the extent to which it is a problem needs further investigation. Interestingly, Broyde et al. (2021) also suggested that inter‐observer variation of fascicle length can lead to differences in force estimates both between and within papers.

As an example, Sustaita (2008) calculated bite forces for birds of prey that were higher than measured bites forces (Sustaita & Hertel, 2010). For the Peregrine falcon (*Falco peregrinus*), bite force was calculated at 16.9 (±2.9) N but in vivo measurements recorded a lower bite force of 12.96 (±1.84) N; whether this reflects muscle shrinkage is unclear. In vivo bite forces were also lower than predicted by biomechanical modelling by Becerra et al. (2011) on the rodent Talas tuco‐tuco (*Ctenomys talarum*). In both cases, the authors concluded that perhaps the animals were not using the maximal force they could generate due to different motivational factors. However, it could be that the process of isolating the ‘fascicle’ introduced shrinkage of the muscle reducing the length of the fascicle.

In this study, fascicle length determined from dissected muscles produced a low value for bite force when compared to a published value (Carril et al., 2015; Cost et al., 2020; Dickinson et al., 2022) or measured results for parrots. Only when the morphology of the muscle was considered in situ and fascicle lengths were determined using area values determined, were bite values comparable to measured values. This method of determining fascicle length was novel but was validated by empirical values measured for live parrots. It is important that the muscles were studied using images of them in situ because this was more physiologically and morphologically realistic. However, it is appreciated that further research is required to validate this method for various other species.

### 4.4 Inferences for the predictions of bite force

Physical elements of the skull allow for prediction of a functional trait, such as skull width being a reliable estimator of bite force in finches (van der Meij & Bout, 2008). Although in the fossil record soft tissues are often not preserved (Lautenschlager et al., 2013), other osteological elements, such as muscle scarring, are commonly measured as a proxy for the size of muscles (Sakamoto et al., 2019; Wroe et al., 2005). This ‘dry skull method’ and has been extensively used to predict bite force in extinct species, especially mammals (Huber et al., 2005; Law & Mehta, 2019; Thomason, 1991; Walmsley et al., 2013). Alternatively, Sakamoto (2022) used skull length and phylogeny to predict physiological cross‐sectional area of muscles to predict bite force in dinosaurs. Bite force in extinct archosaurs has also been estimated by calculating the area of muscle coverage between attachment and insertion points and using this area to calculate the force of the muscle (Pêgas et al., 2021). These methods typically require many measurements (depending on the species of interest) and assume that the morphology of jaw muscles is comparable between extant and extinct species. Here, we showed that the length of the *m. adductor mandibulae* scar allowed accurate prediction of bite force in parrots. However, use of this muscle scar may not directly applicable to other bird orders because of variation in the jaw musculature. It would be interesting to repeat the present study for other bird orders to assess whether similar relationships exist using a similar mandible measurement.

The relationship between bone tissue and muscle tissue was not always isometric (Harrison et al., 2022) making prediction of soft tissue elements more complicated. The fossil record is predominantly comprised of hard tissue remains (Bates & Falkingham, 2012; Cox et al., 2015; Viglietti et al., 2020), which are used to make predictions on different aspects of the specimen's ecology (Pêgas et al., 2021). For example, for the terror bird (*Andalgalornis steulleti*) of South America, Degrange et al. (2010) suggested that the beak was not as structurally solid as observed in extant predatory birds, and its estimated bite force of 133 N, extrapolated from estimated body mass, indicated a diet of small animals despite the skull being 370 mm long. However, Deeming et al. (2022) suggested that the different groups of birds may exhibit different relationships between elements which are used to determine bite forces and perhaps using data from finches to predict bite force in extinct raptors may not be appropriate. This highlights the importance of incorporating soft tissue comparisons between extinct and (if possible) related extant species when modelling a function which requires the given tissues input.

Additionally it could be argued that the way in which we generalise muscle arrangements and properties needs to be re‐assessed. For example, many studies use the isometric force value of 0.25–0.30 N/mm^2^ when calculating the maximal force generated by a muscle (see review by Martin et al., 2020). However, these values were retrieved from skeletal muscles of guinea pigs and rats (Lieber & Fridén, 2000; Medler, 2002). It is possible that the muscles surrounding the jaw, which are broader and attach along the surface of the bone, possess different tensile properties. Further study into the maximum tetanic stress values for different muscle types in various vertebrates would be beneficial for modelling jaw biomechanics. Previous studies have shown that stress values can indeed vary greatly across taxa ranging from 20 to 40 N/cm^2^ (e.g. Herrel et al., 2007; van Wassenbergh et al., 2007).

### 4.5 Bite force, feeding behaviours and diet

We acknowledge that the model used here was a simple estimation of bite force in parrots. Parrots are highly intelligent (Emery, 2006) and possess a wide array of behaviours involving their jaws, including, and not limited to, climbing and locomotion (Demery et al., 2011; Dickinson et al., 2024; Young et al., 2023), preening (Bush & Clayton, 2018), and manipulation of objects (Auersperg et al., 2012). Therefore, limiting calculation of the bite force distribution to only one point, whilst provides empirically consistent bite force values, may be an underestimation of force generated by the jaws under different circumstances. Additionally, the ability to move the upper jaw Independently of the lower jaw (cranial kinesis) may structurally provide a system in which the jaw compresses both downwards and upwards applying force from both directions (Hoese & Westneat, 1996; Krishnan, 2023; Mahmoud et al., 2017). However, the muscles which are involved in manipulation of the upper mandible were included in the bite force estimates, but due to the complexity of the muscle arrangements, they were treated as one complex of muscle and so the role which they play in cranial kinesis was unaccounted for in this model.

When considering bite force in relation to diet, the bite force generated in parrots may be insufficient to process common food items. Schüler et al. (2014) estimated that maximal fracture force required to break a macadamia nut (*Macadamia integrifolia*) was around 3900 N. However, fortunately for parrots, other seeds measured typically required less force to crack, for example, almonds (*Prunus dulcis*) require around 1200 N maximal force and peanuts (*Arachis hypogaea*) only 117 N. The maximal force required to crack a macadamia nut may be higher than the measured value for a macaw bite but they can still process these nuts (Gilardi & Toft, 2012). Cracking a macadamia nut by the hyacinth macaw involves the roof of its mouth (covered by projections called the choanal papillae) to shell the nut of its outer coating before further mastication of the kernel (Collar, 1997; Martens et al., 2013). This continual chiselling motion, using the tip of the lower jaw to push the food item against its palate requires micro‐movements of the jaw (Bright et al., 2019). It is probable that repeated loading also causes crack propagation in the seed's testa that will result in much lower force values required to crack the nut, but a more detailed analysis of the processing of nuts by parrots is needed to confirm this.

Unlike the parrots, finches process durable food types by positioning the object between its jaws using its tongue, then they apply force along the ‘seams’ of the shell until it cracks (Andries et al., 2023; Heckeberg et al., 2021; Mielke & van Wassenbergh, 2022; van der Meij et al., 2004). Additionally, the food item is usually positioned proximally where it is crushed between the blunt tominal edge of the beak and the ridge of the maxilla, and then it is cut open through microshearing movements of the beaks (Grant, 1981; Nuijens & Zweers, 1997; van der Meij et al., 2004; van der Meij & Bout, 2004), which requires specific skills in the precise movement of the beak (Andries et al., 2023; Mielke & Van Wassenbergh, 2022). van der Meij et al. (2004) showed that the Eurasian greenfinch (*Carduelis chloris*) was more efficient at cracking seeds in relation to other seed eaters, such as Java sparrows (*Padda oryzivora*) and Yellow‐fronted canaries (*Serinus mozambiques*), because of its wider husking groove that allows it to properly stabilise the food item before applying pressure. This means fewer cracking attempts because the force can be continually applied to the seed with a lower risk of seed displacement during husking. By contrast, parrots appear to favour the use of the lower jaw and their feet to manipulate the food item between the more distal edge of the lower mandible, pushing the food item against the maxilla's palate, which is lined with ridges providing an uneven surface to chisel the seeds and nuts against, aiding in de‐husking (Bright et al., 2019; Homberger, 2003; Martens et al., 2013).

In finches, the relatively simple means of food preparation requires high levels of force and is reflected in the positively allometric relationship observed between finches' body mass and bite force capability (van der Meij & Bout, 2004). Comparison of the slopes generated here show that whilst parrots have the highest estimated bite forces amongst birds (see also Dickinson et al., 2022), the relationship between body mass and bite forces scaled at a higher rate within finches (slopes of 1.26 and 1.55 for Estrilididae and Fringillidae, respectively). Scaling a finch to the same body mass as a macaw means that they would generate larger bite forces (Sakamoto et al., 2019). For example, the large ground finch (*Geospiza magnirostris*), which weighs only 33 g, had a maximal bite force of 70 N, which gives a bite force to body mass value (BF:BM) of 2.12. This is 4.3 times that of the highest BF:BM value calculated for a parrot reported here (Scarlet macaw [*A. macao*] at 0.44). Therefore, *G. magnirostris* possesses the highest bite force in proportion to its body mass.

Interestingly, the hawfinch (*Coccothraustes coccothraustes*) has an *m. ethmomandibularis* (Sims, 1955), which to the best of our knowledge is only found in parrots. There is no estimate for bite force in this species (van der Meij et al., 2004) but the closely related Grosbeaks yellow billed grosbeak (*Eophona migratoria*) and the Collared grosbeak (*Mycerobas affinis*) have bite forces of 36 and 38 N (van der Meij & Bout, 2004) and BF:BM ratios of 0.69 and 0.54, respectively. Due to the similarity in jaw muscle masses, it is expected that the hawfinch has a bite force similar to these grosbeak species but if the *m. ethmomandibularis* is only present in the hawfinch the bite force may be higher. Further research is needed to document whether grosbeaks also possess this muscle and to compare the measured bite force of the Hawfinch to other finches and also compare it to a similar sized parrot, such as the rosy‐faced lovebird (Dickinson et al., 2022), to see how much the differences in jaw musculature impact on bite force.

These acts of processing the food prior to ingestion in these different groups of birds may be the bridge between maximal bite force and minimal fracture force for the food item. This is an interesting concurrence as it highlights the idea that the physiological features of an organism do not act in isolation; there is an interplay between the physiology and behaviour of an animal which needs to be considered and would be a fruitful area of research in birds and other vertebrates.

## 5 CONCLUSIONS

Skeletal and soft tissue components of the skull in psittaciforms exhibit different relationships with an apparent de‐coupling having occurred between the two, this relationship needs to be considered when examining soft or hard tissues in the absence of the other. Bite force in parrots can be accurately predicted using the thickness of the muscle in PCSA measurements, as corroborated by empirical bite force values measured using a force transducer. These forces in large parrots represent the highest bite force calculated in birds to date and because force scaled isometrically with body size, suggesting that bite force is conserved in this order. Through the use of length measurements on the *m. adductor mandibulae* scar along the lower jaw it was possible to predict bite force in parrots. This means that it could be possible to measure the scar from the skulls of other parrot species and predict bite force without recourse to muscle dissection.

## AUTHOR CONTRIBUTIONS

DCD, GPS and SLH conceived the ideas and designed methodology; SLH and AH collected the data; DCD, GPS and SLH analysed the data; DCD and SLH led the writing of the manuscript. All authors contributed critically to the drafts and gave final approval for publication. Many thanks to reviewers who help to improve previous iterations of this manuscript.

## CONFLICT OF INTEREST STATEMENT

The authors declare no conflict of interest.

## Data Availability

Data openly available in a public repository that does not issue DOIs.
